# Embedded implementation research in programming at scale – the new normal to be!

**DOI:** 10.1186/s12887-023-04470-8

**Published:** 2024-02-28

**Authors:** Stefan Swartling Peterson

**Affiliations:** 1 Karolinska Institutet and Uppsala University, Stockholm, Sweden; 2 Makerere University, Kampala, Uganda

**Keywords:** Implementation research, Know-do gap, Capacity development

## Abstract

If you want to run faster, don’t just buy a new pair of shoes; also consider your training methods and where you run.

This supplement examines six countries that have run faster than others in reducing under-five mortality, taking an implementation research approach, with country case studies done with local researchers and local institutions. Key generalizable learnings are to choose and adapt implementation strategies to context, design strategies to target the most vulnerable, systematically learn from implementation experience, and to leverage non-health-sector contributions.

Embedding implementation research in programming has the potential to greatly improve and accelerate the contextualization and implementation of evidence-based child survival interventions to improve equity in coverage and overall effectiveness in reducing under-five mortality. It is now time to build such capacity in local institutions at scale, and incentives for concerned stakeholders to make this the new normal. Regional institutions should now take the lead in making this happen, not just in individual institutions and countries, but across entire regions, supported by global partners.

**Trial registration **N/A.

## Main text

I got myself a new pair of running shoes. The latest and best. But I still do not run faster, further, or even more frequently…Should I change shoes? My family suggests I ought to concentrate on the training strategies rather than the shoes. And that I study how I train, and adapt the training methods to my age, body mass index, and to my progress, by learning from how I fare in my training. They even propose I should think about what I am eating and how I sleep! Could they have a point?

In the health sector we take a lot of interest, and do a lot of research, to develop improved evidence-based interventions, new “shoes”, as it were. But rarely do we take a structured interest, and apply scientific methods, to improving and contextualizing the delivery of those same interventions, the “training” in my analogy. Nor do we necessarily bother to look at the contributions from context and other sectors to health outcomes.

The papers in this supplement do just that. They start from six countries that have done exceptionally well in reducing under-five mortality (U5M) and examine why they are “exemplars.” Using an implementation science framework with five steps: exploration, preparation, implementation, adaptation, and sustainment, they examine tracer evidence-based interventions using a mixed methods approach. They also look at the context, and how it helped or hindered progress.

Cross-country [1] and equity analysis [2] papers find that adapting implementation strategies to context, designing strategies to target the most vulnerable, systematically learning from implementation experience, and leveraging non-health-sector contributions are important generalizable learnings.

A key finding is the importance of in-country research institutions that can work with local policymakers, implementers, and communities to learn from programming, and contextualize and validate findings from elsewhere, as recently called for by e.g. Rasanatan and Ghaffar [3, 4]. In the studied countries this took different forms, e.g. in building implementation research capacity within a Ministry of Health [Rwanda), or working with a local university (Senegal) or research organization (Bangladesh) [1].

Lessons learnt by in-country researchers include the importance of accountable leadership and community-based health systems as favorable contexts from Bangladesh, and an increase in national budget and integrating interventions into health systems as key strategies. Ethiopia’s Health Extension Program, and decentralization of the health system, and subnational contextualization served to reduce inequities in access and U5M. Nepal findings point to the use of local research to guide policy in the expansion of community- as well as facility-based approaches with strong community engagement. From Peru the strong focus on equity in service coverage stands out, and from Rwanda continuous monitoring, evidence-based management, and a participatory approach.

It is therefore high time that capacity building in individuals and institutions in implementation research is seen as essential parts of countries’ human resource strategies. Training models exist (see for example TDR’s SORT IT, (https://tdr.who.int/activities/sort-it-operational-research-and-training)), as do individual centers of excellence. But what is needed is scale! Here the rapid establishment by the Africa CDC of regional networks for COVID across Africa, based on individual centers of excellence, is very encouraging [5]. In a next phase, may I suggest Africa CDC and its regional counterparts, supported by development banks, donors, and national governments, take on such capacity development in implementation research at scale, across their regions [6].

Building an ecosystem where embedded research in programming is the new normal will also require changing and building incentives. For programmers and funders, to add implementation research and its methods toolkit to the monitoring and evaluation budget-line, which will not just help more programs and countries become “exemplars,” but also support local researchers to do this type of work [3, 4, 6]. And for research institutions, to value work that does not just produce high publication impact factors in prestigious international scientific journals, but actually changes policy and practice locally through locally relevant research, which engages the key stakeholders in co-production [6]. This may also serve to redefine global health, when the primary scene becomes local [7], and that when and if you publish internationally, the local author is naturally the primary author and partnerships more equal [8].

So, what did I learn for myself? Well, I have concluded that it’s not the model of my shoes that is the rate-limiting step to my running. I will have to look into whether I train (adoption, fidelity) and how I train (appropriateness), where I run, the weather, and what I eat (context), and apply a learning from practice approach.

## Conclusions

Embedding implementation research in programming has the potential to greatly improve the contextualization and implementation of evidence-based child survival interventions to improve equity in coverage and overall effectiveness in reducing under-five mortality. It is now time to build such capacity in local institutions at scale, and build incentives for concerned stakeholders to make this the new normal.

## Data Availability

Not applicable.

## Abbreviations

CDC: Centers for Disease Control and Prevention
U5M: Under-5 mortality
